# Analysis of Fibropapillomatosis in Roe Deer (*Capreolus capreolus*) Confirms High Content of Heavy Metals

**DOI:** 10.3390/ani14192847

**Published:** 2024-10-03

**Authors:** Klára Matějka Košinová, Jan Cukor, Vlastimil Skoták, Rostislav Linda, Zdeněk Vacek, Karel Bukovjan, Tomáš Kušta

**Affiliations:** 1 Faculty of Forestry and Wood Sciences, Czech University of Life Sciences Prague, Kamýcká 129, 165 00 Prague, Czech Republic; cukor@fld.czu.cz (J.C.); vacekz@fld.czu.cz (Z.V.); kusta@fld.czu.cz (T.K.); 2 Forestry & Game Management Research Institute, Strnady 136, 252 02 Jíloviště, Czech Republic; skotak@vulhm.cz (V.S.); rostislav.linda@live.com (R.L.);; 3 Faculty of Forestry and Wood Technology, Mendel University in Brno, Zemědělská 1, 613 00 Brno, Czech Republic

**Keywords:** deer health status, game management, heavy metal toxicity, meat quality, skin tumour, wildlife disease

## Abstract

**Simple Summary:**

In Central Europe, one of the main issues in wildlife management at present is its increasing abundance. As population density increases, diseases that directly or indirectly affect humans are also becoming more prevalent. Although fibropapillomatosis is one of the diseases that does not threaten humans, the accumulation of some heavy metals in tumours has been shown to occur at concentrations that would already be toxic to humans and wildlife. In general, the heavy metal content in wild animal tissues is a partly known topic that was studied on muscle or internal organs but is almost unstudied in tumours. Therefore, we evaluated the content of selected heavy metals in roe deer—the most widespread wildlife species across Europe. If the accumulation of these heavy metals in the tumours also affects the muscle of the individual, which is then intended for consumption by the final consumer, such meat could be considered a health hazard. This pilot study is a cornerstone for further research to clarify the safety or otherwise of meat from wild game affected by fibropapillomatosis.

**Abstract:**

In recent decades, there has been an increase in European wild ungulate populations, often associated with a decline in health and spread of disease. This is true for the roe deer (*Capreolus capreolus*), the most common European cervid, with populations apparently affected by fibropapillomatosis, an increasingly common cancer. To date, however, there has been little research into this disease, thus many interactions remain unclear and descriptions of tumour composition are poorly validated. The main aim of the present study was to evaluate the presence and concentration of toxic heavy metals in roe deer skin tumours. Our results confirmed the presence of virtually all the metals tested for, i.e., Pb, Hg, Cd, As, Cr, Mn, Al, Co, Cu, Ni, Se, Zn, and Fe, with the highest average concentrations found for Cr (0.99 mg/kg^−1^ ± 2.23 SD), Cd (0.03 mg/kg^−1^ ± 0.03 SD), and Hg (0.02 mg/kg^−1^ ± 0.02 SD), exceeding FAO limits for meat from slaughtered animals. We also observed a significant positive relationship between heavy metal concentration and age, especially for Pb, As, Hg, Mn, Se, Al, Zn, and Ni. Our findings provide a strong baseline for further research on the impact of fibropapillomatosis, not only on the welfare and health status of game but also on the final consumer of venison, which in many respects is regarded as a high-quality, ecological, and renewable wild resource. While deer with this disease are not considered qualitatively or medically defective, they could represent a potential reservoir of substances toxic to humans and could affect substance levels in adjacent tissues or the animal as a whole.

## 1. Introduction

An increase in wild ungulate abundance in Central Europe over recent decades has become a main topic of research on large mammal populations [1,2,3,4,5,6,7,8,9]. In addition to their potential to adversely affect cultural landscapes and forest ecosystems [8,10,11], high population densities increase the chances of human-wildlife conflicts and increase the risk of disease spread [9]. This is also why the concept of overpopulation of wild ungulates is now frequently invoked in the context of population increases [8,9]. To determine whether populations are ‘overbreeding’, biologists monitor a series of biological criteria [8], and where such criteria indicate the fitness of a species is being affected, the term overpopulation can be applied [12].

As an example, significant population growth has been recorded over recent decades in the Central European population of roe deer (*Capreolus capreolus* L.), as demonstrated through the reports of hunted game, which are considered to be the most conclusive hunting statistics data [13]. As this game species is highly territorial [14], high population densities lead to intraspecific stress [15], which can lead to impaired physical condition [7], an important factor affecting possible disease resistance [16]. Evidence of the poor physical fitness and health status of European roe deer populations can be illustrated using data from the Czech Republic, where a total of 114,100 roe deer were hunted over the 2022/23 hunting season, but a further 50,365 were reported as found dead [17]. This relatively high number of dead animals suggests that the deer may be in poor physical condition and that the incidence of disease in the population may have increased accordingly.

A disease that often accompanies such increases in roe deer population density is fibropapillomatosis [18,19,20]. In addition to cervids [18,21,22,23,24], this viral disease is known to affect antelope, zebra, buffalo, giraffe [25,26,27], camels [28], rabbits [29], and some carnivorous species, including the American cougar (*Puma concolor cougar*) [30], as well as domestic animals such as horses and cattle [31]. The typical feature indicating the presence of the fibropapilloma virus is the formation of skin tumours of different size, location, frequency, coloration, and type. In terms of histopathology, it is a dense tissue of fibroblasts/fibrocytes and collagen fibres clearly separated from normal skin [32]. They are benign tumours that are most commonly present in two basic forms, sessile and pedunculated [24]. A new species of the papillomavirus, CcPV1 [33], has been recognised as the causative agent of fibropapillomatosis in roe deer by the International Committee on Taxonomy of Viruses [34].

The primary mode of transmission of the disease is through contact between infected and healthy individuals, specifically contact between infected tissue and disturbed tissue of a susceptible individual [24,35]. Transmission through vegetation or other contaminated material is also likely, as such transmission has been confirmed for turiform papillomavirus [31]. There is also speculation on transmission by stinging insects, with DNA sequences of both CcPV1 and CePV1 having been confirmed in ticks collected from infected individuals and from samples collected from hunting dogs [32]. Documentation on the occurrence of fibropapillomatosis has so far been limited mainly to Central Europe. In the Czech Republic, for example, the first occurrence was documented in 2007 [36], since when there has been a constant spread of the disease. By 2017, the disease had been recorded and confirmed in 1549 hunted roe deer from four of fourteen regions [37]. In contrast, the first case in Slovakia had been recorded as early as 1998, though only 454 cases had been documented by 2014. Cases are also known from other European countries, including Italy [38], Croatia [39], Spain, France, Hungary, England [33], and Slovenia [21,40].

To date, the composition of fibropapillomatosis tumours has not been comprehensively described. While histopathological analyses are known, previous studies have tended to focus on selected substances only, such as metallothioneins, taurine, or zinc [32]. In the latter study, the authors reported a metallothionein content of 2.6 mg/g^−1^ in control skin samples from healthy individuals and healthy skin samples from infected individuals, a concentration, on average, 20–30% lower than that of papilloma samples (though based on a low number of analyses). Metallothioneins are known to bind heavy metals [41], such as mercury (Hg), and it is likely that it is the presence of metallothioneins that increases the concentration of heavy metals at the papilloma site and possibly in the organs or flesh. This could lead to negative impacts on wildlife and potentially also humans through the food chain.

Heavy metals, such as zinc (Zn), are found throughout the environment in varying concentrations, both in vegetation and in the tissues of domesticated and wild animals. The amount of heavy metals in tissues may be subject to seasonal fluctuations, reflecting the influence of environmental conditions, weather conditions, and therefore available food [42]. Seasonal peaks in metal concentrations have been reported in different ways, with some studies showing the highest concentrations during August to September [42], others during winter [43] or spring to summer [44]. While some of these metals are essential in small doses for the body’s metabolism [45], at higher levels they can have adverse side-effects and be harmful to both humans and animals [46]. Heavy metal toxicity is evaluated based on the overall harmfulness and severity of the effect [47], with negative effects varying by element. Concentrations accumulated in the body will depend on the dose consumed and the duration of exposure, as well as the type of organism and its age [48]. The presence of a number of heavy metals, including chromium (Cr) and nickel (Ni), has been associated with some cancers [49], while arsenic (As) has been shown to cause cardiovascular disease, kidney failure, neurotoxicity, diabetes, hepatotoxicity, hypertension, and various cancers following prolonged exposure [50]. Cadmium (Cd) occurs naturally in the Earth’s crust at very low concentrations [51]; however, the element is highly toxic and can have adverse effects on both the environment and human health, being often associated with cases of cancer [52]. Finally, lead (Pb) has been shown to increase blood pressure, slow nervous system functioning, and cause drowsiness, concentration disorders, fertility disorders, headaches, and, in severe cases, encephalopathy or death in adult humans [53,54], with similar negative impacts also confirmed in animals.

One might assume, therefore, that heavy metals are likely to be present in CcPV1-derived tumours [32]. However, the presence of these substances has yet to be described, despite their negative impact on human [53,54] and animal health [55,56] having been demonstrated. Owing to their rate of growth and spread, skin tumours could potentially be an important indirect source of health complications alongside the tumour, not just as they may serve as a reservoir of heavy metals but also because these metals could affect surrounding tissues or even the whole body. This is a very complex issue, as insufficient regulation of game numbers has a noticeable impact on the environment but at the same time does not benefit the population itself. High concentrations of game create favourable conditions for stress, with the consequent deterioration in physical condition and, consequently, the easy spread of diseases, both those transmissible to humans and those that may not at first sight pose a direct threat. Unfortunately, some diseases may have unexplained aspects that are dangerous and health-threatening to humans.

The aim of this study, therefore, was to (i) verify the presence of selected heavy metals in skin tumours, focusing on those with known high toxicity; (ii) assess whether the presence and concentration of each substance are dependent on the sex of the individual; and (iii) define to what extent the ratio of substances and their concentration depends on the age of the infected individual and the type of tumour (i.e., pedunculate or sessile).

## 2. Material and Methods

### 2.1. Study Area

**Figure 1:**
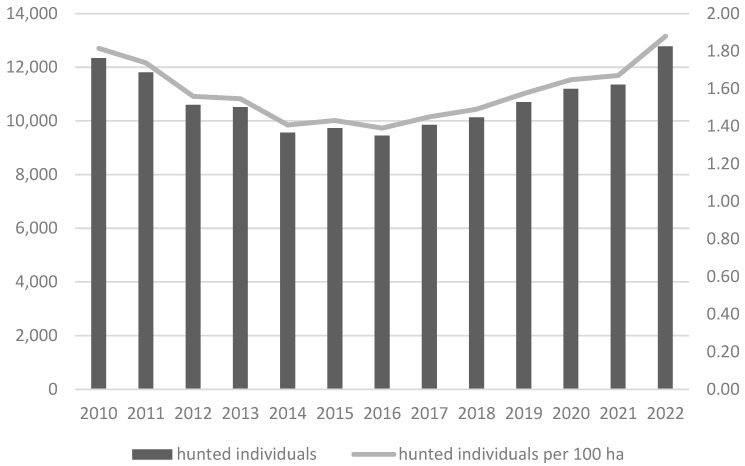
Roe deer (*Capreolus capreolus*) hunting bag for the Vysočina Region (Czech Republic) between 2010 and 2022 [17].

Skin tumour samples were obtained from infected roe deer from the Vysočina region of the Czech Republic, one of the first areas in Europe with confirmed occurrence of fibropapillomatosis in roe deer. Situated in the central part of the Republic, the sample area covers 6 796 km^2^, of which 1.8% is water. Temperatures range between 6–8 °C and annual rainfall between 500–800 mm. The landscape is characterised by spruce forest communities (77% of total area, 10% pine, 4.6% beech, 3.1% larch, 2.5% oak, and 2.8% other tree species) and a large number of small water bodies, mostly in the form of artificial fish ponds, with an additional 3% of the total area comprising peat bogs, 1% other waterbodies, 2% wetland and riparian communities, and 9% by arid to wet meadow communities, these often forming elements of nature monuments and reserves [57]. In the 2022/23 hunting year, a total of 12,772 roe deer (1.9 ind/ha) were hunted within the Vysočina Region, the third highest number for all regions of the Czech Republic (Figure 1) [17].

### 2.2. Sample Collection

Between 2010 and 2024, whole tumours were collected in the field from a total of 76 roe deer legally hunted by regional hunting organisations according to Act No. 449/2001 Coll. The deer ranged from one year to eight years of age (age estimated based on dentition), and sex distribution was almost even, comprising 37 females and 39 males. The skin tumours were divided into two categories according to type of growth, i.e., sessile or pedunculate, then frozen and sent to the laboratory for further analysis.

### 2.3. Laboratory Analysis

A total of 13 metals were assessed in this study, i.e., lead Pb, mercury Hg, cadmium Cd, arsenic As, chromium Cr, manganese (Mn), aluminium (Al), cobalt (Co), copper (Cu), Ni, selenium (Se), Zn, and iron (Fe). Prior to analysis, each whole skin tumour was first homogenised using a laboratory grinder. The abundance and proportion of Hg in skin tumours was determined by atomic absorption spectrometry [58], while Pb, Cd, As, Cr, Mn, Al, Co, Cu, Se, Ni, Zn, and Fe were determined using inductively coupled plasma spectroscopy [59]. For limits of detection (LOD), see Table 1.

### 2.4. Statistical Analysis

In all cases, readings below the LOD were converted to 0 prior to statistical analysis. For all elements except Pb and As (see below), dependence of element concentration on age was assessed using linear regression, with dependence type confirmed when the slope of the regression line was significantly different from 0. Linear regression predictions were based on the observed range of age values (i.e., the regression lines do not pass through the axis origin for zero age). Dependence of element concentration on the sex of the individual was assessed using the Wilcoxon test, as was dependence of element concentration on the type of papilloma (in each case, the assumptions for the t-test (i.e., normality of data variation) were not met).

For Pb and As, many values were below or at the LOD; hence, the concentration data for these elements were altered to “detected” and “not detected.” In this case, dependence of detection on age was assessed using the Wilcoxon test (age of individuals with element detected vs. age with element not detected), while dependence of element detection on sex and type of papilloma was assessed using Fisher’s exact test.

All data analyses were performed in the R software, version 4.4.0 environment [60], with linear regression graphs created using the R package “ggplot2.” A significance level of alpha 0.05 was considered for all analyses.

## 3. Results

### 3.1. Representation of Metals in Skin Tumours

Metal concentrations showed no significant differences between sampling years. Most of the individuals tested contained most or all of the metals tested for (see Table 2). For example, Hg and Ni were detected in 69 of the 76 deer, while Cd and Se were found in 74 of the 76 deer (both negative samples from a deer negative for Ni). The highest concentrations were recorded for Fe (6.38–36.81 mg/kg^−1^) and Zn (3.66–9.64 mg/kg^−1^). Higher concentrations were also recorded for Cr and Ni, though these were not detected in all samples. In the case of Co, one sample was below the LOD. The lowest concentrations were recorded for As, Hg, Pb, and Cd. For Pb, only 12 samples had concentrations above the LOD, while 64 samples had Pb confirmed but at very low concentrations. A similar situation was also observed for As, where only 11 samples had concentrations above the LOD (Table 2).

### 3.2. The Metal Concentration and Age of the Individual

**Figure 2:**
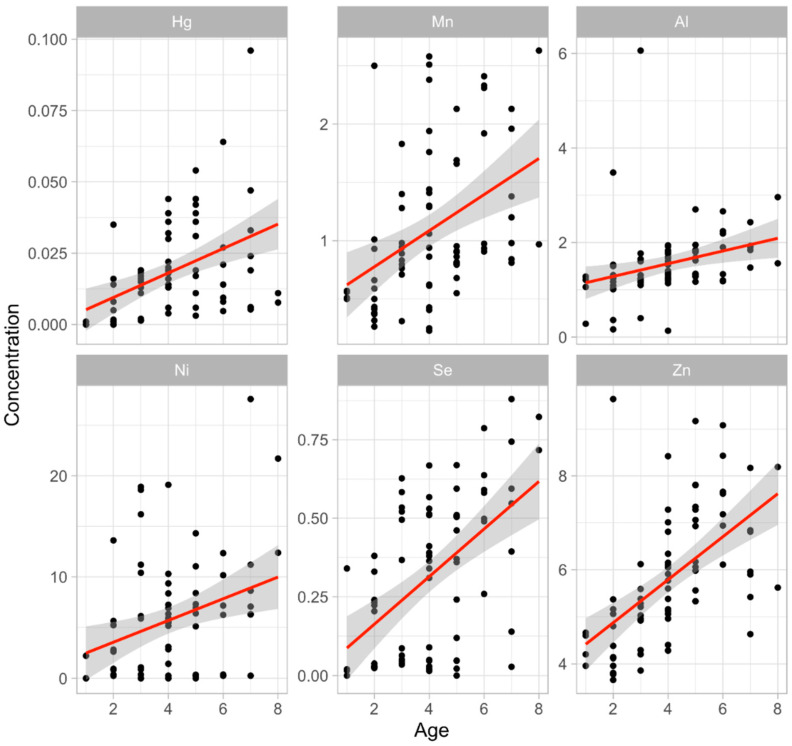
Dependence of Hg, Mn, Al, Ni, Se, and Zn concentration (mg/kg^−1^) on roe deer age.

There was a strongly significant positive correlation between metal concentration and age for Hg (r^2^ = 0.19), Mn (r^2^ = 0.17), Se (r^2^ = 0.28), Zn (r^2^ = 0.32) (all *p* < 0.001), Ni (r^2^ = 0.10), and Al (r^2^ = 0.09) (both *p* < 0.01) (see Figure 2), but no significant relationship for Cd, Cr, Co, Cu, or Fe. There was also a significant positive correlation for Pb and As (*p* < 0.001), the mean age of deer with Pb and As being six years.

### 3.3. Relationship of the Metal Concentration and Sex of the Individual

Significant differences in element concentrations between males and females were observed for Pb (*p* = 0.025) and As (*p* = 0.007; a Fisher exact test was used in both cases). The presence of Pb was recorded in 10 of the 12 positive results, only two of which were females. As showed a similar trend, with 10 of the 11 positive results being male. A significant difference was also observed for Al (Wilcoxon rank-sum test, *p* = 0.02). Insignificant marginally were further observed for Cr (*p* = 0.06), Se (*p* = 0.098), and Zn (*p* = 0.06). While male and female values for Hg, Cd, Co, Cu, and Zn were similar, values for Cr and Fe were noticeably higher (>0.1 mg/kg^−1^) in females, while values for Mn, Al, Ni, and Se were noticeably higher in males (Table 3).

### 3.4. Relationship of the Metal Concentration and Papilloma Type

There was no significant correlation between metal concentration and papilloma type for any of the metals tested. Nevertheless, there were clear differences between concentrations for some substances, the greatest being in the concentration of Fe, where sessile papillomas had an average concentration of 26.415 mg/kg^−1^ and pedunculate papillomas 20.394 mg/kg^−1^ (Table 4). Sessile papillomas also tended to have somewhat higher average concentrations of Cr, Co, and Ni (Table 4).

## 4. Discussion

Metals, including heavy metals, occur naturally in the environment at very low concentrations. Where they are found at elevated concentrations, it is often due to localised pollution, typically occurring in or around large cities or other sites affected by human activity. As many metals are toxic to humans at higher concentrations, often contributing to the development of serious health issues such as cancer or heart disease [61], their presence in food, including domesticated and wild organisms, is under increasing scrutiny. Indeed, concentrations of heavy metals in animal tissues are now frequently used as a bioindicator of environmental pollution [42,62,63]. In the case of game animals such as roe deer, however, it can be difficult to interpret observed values, given the virtual absence of scientific studies on such species. Consequently, in this study, we are forced to compare our results for roe deer with those of other game species.

Several metal concentrations recorded in our roe deer skin tumours were above national or international thresholds set for food sources. In addition to the ‘essential’ metals (i.e., Fe, Cu, Zn, Se, Co, and Mn; needed for bodily functions), previous examinations of papillomas have also confirmed the presence of several toxic metals, including Pb, As, Al, Hg, Cr, Ni, and Cd [53,54], the latter being recognised as one of the most toxic metals recorded in the environment [64]. While a study in Hungary failed to record a measurable presence of Cd in the muscle of roe deer [65], our results showed the presence of Cd in 97.4% of the deer papillomas tested. This may be due to essential differences between Cd uptake in tumours and muscle. This compares well with [63], who reported Cd presence in 84% of their samples of roe deer kidney, liver, and muscle. According to the UN Food and Agricultural Organisation (FAO), the maximum permissible level of Cd in the meat of slaughtered animals is 0.05 mg/kg^−1^ [66], a limit that was only exceeded in nine of 76 samples, i.e., the concentrations recorded in this study were almost all below the maximum permissible value, ranging from 0.005 to 0.2 mg/kg^−1^, values that basically correspond to normal levels in the soil [67]. Using red deer (*Cervus elaphus*), the authors of [68] were able to demonstrate differing Cd concentrations in different tissues, with average concentrations of 0.22 mg/kg^−1^ in muscle, 0.7 mg/kg^−1^ in liver, and 12 mg/kg^−1^ in kidney, the latter concentration potentially high enough to cause pathological changes in the bodies of roe deer [55].

Concentrations of Pb in skin tumours ranged from 0.01 to 0.07 mg/kg^−1^, which places the samples below the European recommended maximum permissible level of Pb in meat of 0.1 mg/kg^−1^ [69] and, as such, the samples do not represent a health risk. Ref. [70] reported significantly lower concentrations, ranging from 0.00028 to 0.00058 mg/kg^−1^, in liver and kidney samples taken from roe deer in Poland, with similar values also recorded for red deer and wild boar (*Sus Scrofa*). On the other hand, significantly higher Pb concentrations, ranging from 0.008 to 8.455 mg/kg^−1^, were recorded in roe deer muscle from Serbia [71], though these values were believed to have been influenced by Pb shot residues.

Only 11 of 76 samples (i.e., 14.5% of total) had detectable levels of As, though at low concentrations ranging from 0.005 to 0.008 mg/kg^−1^. As these concentrations are well below the maximum permissible concentration of As in slaughter meat, i.e., 0.2 mg/kg^−1^ [72], the samples do not represent any threat to human health. Other studies have also recorded low levels of As contamination in game tissue, with [73] finding no samples with As above the LOD with a strong dependence on the sex of the individual in Germany and [74] only finding As residues in 29% of their wild boar tissue samples. Our data showed a strong dependence between As concentration and sex of the individual.

We recorded concentrations of Al ranging from 0.13 to 6.06 mg/kg^−1^ (average 1.57 mg/kg^−1^) in our study, levels that compare well with those of [75], who recorded an average concentration of 3.170 mg/kg^−1^ in the *biceps femoris* muscle of fallow deer (*Dama dama*), and 5.763 mg/kg^−1^ in the *longissimus thoracis* and *lumborum* muscles. The authors of [76] recorded Al concentrations of 0.0006 mg/kg^−1^ in the hair of roe deer, 0.00094 mg/kg^−1^ in the hair of field hare (*Lepus europaeus*), and 0.00181 mg/kg^−1^ in the hair of wild boar, levels significantly lower than those in our study.

Only seven (9%) of our samples (four females, three males) showed measurable levels of Hg, with concentrations ranging from 0.001 to 0.1 mg/kg^−1^. These findings are significantly lower than those recorded by [73], who recorded concentrations of 0.87 mg/kg^−1^ in roe deer muscle. The maximum permissible concentration of Hg in the meat of slaughter animals is set at 0.01 mg/kg^−1^ [66]; thus, some of our values exceed the limits set by WHO and the FAO. In general, Hg concentrations tend to be low in game species, with [77], for example, reporting concentrations of 0.000001 to 0.000005 mg/kg^−1^ in red deer, ref. [78] reporting 0.01 to 0.03 mg/kg^−1^ in European beaver (*Castor fibre*), and [79] recording 0.00001 to 0.00006 mg/kg^−1^ in wild boar, with highest concentrations recorded in the kidney. In contrast, ref. [80] reported concentrations in wild boar below the LOD. A comprehensive study of Hg in meat and organs of wild game in Poland also recorded very low values, with concentrations in muscle ranging from 0.0006 to 0.0056 mg/kg^−1^ and from 0.0008 to 0.0164 mg/kg^−1^ in liver [81]. Ref. [82] reported Hg values similar to our own, recording levels ranging from 0.02 to 0.1 mg/kg^−1^ in muscle, liver, and kidney of wild boar, again with highest values in the kidney. Consequently, our highest values represent some of the highest recorded in European game species.

Significantly elevated values were also recorded for Cr, with concentrations ranging from 0.18 to 19.66 mg/kg^−1^, values significantly higher than the maximum permissible concentration in the meat of slaughter animals at 0.05 mg/kg^−1^ [66]. In contrast, ref. [80] reported an average concentration of only 0.13 to 0.14 mg/kg^−1^ in wild boar muscle, and 0.08 mg/kg^−1^ in fat. In red deer, ref. [83] recorded concentrations of 0.081 mg/kg^−1^ in liver, and 0.156 mg/kg^−1^ in the kidneys, again lower than the values recorded in skin tumours in this study.

The average Ni concentration in our skin tumours was 5.8 mg/kg^−1^ (range 0 to 27.6 mg/kg^−1^), which compares relatively well with the average concentration of 2.8 mg/kg^−1^ (max. 6.7 mg/kg^−1^) recorded in wild boar and roe deer muscle by [84]. On the other hand, ref. [85] reported an average Ni concentration in wild game muscle of 0.000081 mg/kg^−1^, significantly lower than the levels recorded in this study.

The essential metals tested for (i.e., Fe, Cu, Zn, Se, Co, and Mn) occur as components of enzymes and proteins and are essential to health in small concentrations but can manifest toxic reactions at higher concentrations. One such metal that we recorded at elevated concentrations was Fe, with values ranging from 6.38 to 36.81 mg/kg^−1^. These values are significantly higher than the maximum permissible concentration of 0.01 mg/kg^−1^ allowed in meat [72], but significantly lower than the mean Fe concentration in red deer liver of 370.4 mg/kg^−1^ reported by [86]. Likewise [74] reported very high levels in wild boar kidney, with an average concentration of between 91.7 and 171 mg/kg^−1^. Both of these studies were examining organs, however, and, as these are the organs that filter blood, such high concentrations are to be expected. In comparison, values significantly lower than our own for skin tumours were reported by [87], who recorded muscle concentrations of 3.26 mg/kg^−1^ in moose (*Alces alces*), 2.30 mg/kg^−1^ in red deer, 2.06 mg/kg^−1^ in roe deer, and 3.44 mg/kg^−1^ in wild boar. Values somewhat closer to ours were recorded by [75], who reported 38.294 mg/kg^−1^ in the *biceps femoris* of fallow deer and 43.196 mg/kg^−1^ in the *longissimus thoracis* and *lumborum*.

All of the skin tumours in this study contained Cu, with values ranging from 0.06 to 4.36 mg/kg^−1^ (mean 1.13 mg/kg^−1^). These levels are somewhat lower than those recorded in previous studies, with the authors of [71], for example, recording an average concentration of 4.23 mg/kg^−1^ in roe deer muscle but 28.07 mg/kg^−1^ in liver and 15.87 mg/kg^−1^ in kidney. Likewise, ref. [88] recorded an average muscle concentration of 29.68 to 46 mg/kg^−1^.

The values for Zn in this study (3.66 to 9.64 mg/kg^−1^) also exceeded the maximum permissible concentration in meat, which is set at 0.3 to 1 mg/kg^−1^ [72]. Nevertheless, other studies have reported significantly higher values, with the authors of [88], for example, recording concentrations in roe deer muscle of 70.8 to 77.10 mg/kg^−1^, 157.21 to 489.84 mg/kg^−1^ in liver, and even 120.93 to 376.8 mg/kg^−1^ in hair. Ref. [68] reported an average concentration in red deer muscle of 150 mg/kg^−1^, while [80] recorded 52.12 to 56.75 mg/kg^−1^ in wild boar muscle. Generally speaking, Zn causes few problems as it is easily metabolised; however, toxicosis can occur due to the formation of caustic Zn salts, which can cause problems in the digestive tract [89].

The average Se concentration in our study was 0.32 mg/kg^−1^, which is below the maximum permissible concentration in meat of 0.5 mg/kg^−1^ [72]. This corresponds with the results of [90], who reported an average liver Se concentration of 0.32 mg/kg^−1^ in wild boar. The same study, however, reported lower concentrations in red deer (0.08 mg/kg^−1^) and red fox (*Vulpes vulpes*; 0.14 mg/kg^−1^).

Tumour concentrations for Co in our study ranged from 0.05 to 2.4 mg/kg^−1^, with a mean value of 0.33 mg/kg^−1^, values similar to those recorded by [83] in red deer liver (0.361 mg/kg^−1^) and kidney (0.256 mg/kg^−1^). The authors of [79], however, recorded much lower mean concentrations in wild boar liver, kidney, and muscle at 0.000438 mg/kg^−1^, while [68] recorded levels of 0.017, 0.089, and 0.18 mg/kg^−1^, respectively, in red deer muscle, kidney, and liver. In contrast, [82] reported an average concentration of 0.41 mg/kg^−1^ in wild boar muscle and 0.48 mg/kg^−1^ in liver and kidney.

Concentrations of Mn in our study ranged from 0.23 to 2.63 mg/kg^−1^, levels similar to those recorded by [80] for wild boar muscle (male = 1.36 mg/kg^−1^, female = 0.45 mg/kg^−1^) and in fat (male = 0.74 mg/kg^−1^, female = 0.32 mg/kg^−1^). Likewise, ref. [91] reported average Mn concentrations in red deer of 3.47 mg/kg^−1^ in liver, 1.34 mg/kg^−1^ in kidney, and 2.03 mg/kg^−1^ in muscle, with only the concentrations for liver being significantly higher. In contrast, [68] recorded higher values in red deer, with average concentrations in muscle of 2.3 mg/kg^−1^, 12 mg/kg^−1^ in liver, and 6.6 mg/kg^−1^ in kidney.

In this study, age proved to be an important factor influencing the concentration of metals in skin tumours, with older individuals generally providing samples with higher metal concentrations. It is generally known that heavy metal concentrations in tissues are influenced by time of exposure, often associated with age, as well as other explanatory factors such as the sex of the individual or the region from which it originated [63,92]. In our case, age had a significant effect on concentrations of Pb, As, Hg, Mn, Se, Al, Zn, and Ni, with the highest values for Cd, Zn, and Cu found in three- to four-year-old deer. This may be because younger deer are better able to metabolise heavy metals, with levels increasing as the ability to metabolise decreases with age [93]. Just such an increase in metal concentrations as a function of roe deer age has been reported for Cd [55] and for Pb and Zn in the muscle and liver of roe deer in Poland [88]. On the other hand, the latter authors also noted a decreasing trend for Pb in hair samples.

We were able to demonstrate a significant effect of sex on just two of the 13 metals tested, i.e., As and Pb. While male and female values for Hg, Cd, Co, Cu, and Zn were similar, values for Cr and Fe were noticeably higher (>0.1 mg/kg^−1^) in females, while values for Mn, Al, Ni, and Se were noticeably higher in males. If we compare only the occurrence of metals, they were equally more frequent in males than females. Ref. [73] found a similar trend but for Hg in roe deer muscle, with concentrations significantly higher in males than females. Ref. [63], on the other hand, reported that concentrations of Pb, Cd, and Zn were usually higher in female roe deer, as did [88], who also found mostly non-significant differences for Cu. Our results indicate only a higher frequency of some metals but not a difference in concentration.

Some heavy metals, as mentioned, are naturally occurring in the environment, in minerals, soil, or water [94,95]. Wildlife is in close contact with almost all components of the environment, and thus accumulation of heavy metals is expected. Most commonly, these metals enter the the body of wildlife through food and water sources [96]. The presence of higher concentrations of heavy metals in skin tumours may have a negative effect on the individual itself. Poor physical condition, delayed poisoning, and organ diseases—for example, kidney disease in roe deer in association with increasing Cd concentrations [55] or in reproductive cells of European fallow deer affected specifically by heavy metal pollution [56]—are all known manifestations of heavy metal poisoning. Heavy metals in the meat of wild animals pose a certain risk to consumers [83], and fibropapillomatosis in association with heavy metals can bring significant complications, not only for humans but also for the roe deer population itself.

## 5. Conclusions

To the best of our knowledge, this is the first study to address heavy metal concentrations in fibropapillomatosis skin tumours in a game species. At present, the spread of this disease appears to be slow, and regulation of affected individuals through hunting has, so far, been low. The data provided by this study provide a solid basis for further research into the impact of this disease, not only on the welfare and health of roe deer but also on the effects to the final consumer of game meat, which is widely regarded as a high-quality, ecological, and renewable food resource. Our results suggest that skin tumours may harbour a higher concentration of several toxic metals excreted by the body, in the same way that the highest concentrations are usually found in the kidneys or liver, which filter waste substances from the blood. As such, future studies will be needed to assess whether the higher concentrations localised at such tumours, and especially those in severely affected animals, influence levels in adjacent muscle tissue and whether this represents a potential threat to the end consumer, i.e., humans, by analysing tumour tissue and muscle from both infected and healthy individuals.

## Figures and Tables

**Table 1:** Limits of detection (LOD) for the 13 metals examined in this study (mg/kg^−1^).

	Hg	Pb	Cd	As	Cr	Mn	Al
LOD	0.001	0.01	0.001	0.005	0.001	0.01	0.01
	**Co**	**Cu**	**Ni**	**Se**	**Zn**	**Fe**	
LOD	0.05	0.05	0.05	0.01	0.001	0.0001	

**Table 2:** Mean concentration (mg/kg^−1^) of each metal in roe deer tumour samples. N (number of samples tested) = 76.

Metal	Mean	Minimum	Maximum	SD
Hg	0.02	0.001	0.1	0.02
Pb	0.01	0.01	0.07	0.01
Cd	0.03	0	0.2	0.03
As	0.01	0.01	0.01	0
Cr	0.99	0.18	19.6	2.23
Mn	1.1	0.23	2.63	0.65
Al	1.57	0.13	6.06	0.77
Co	0.33	0.05	2.4	0.51
Cu	1.13	0.06	4.36	0.78
Ni	5.8	0	27.6	5.91
Se	0.32	0	0.88	0.25
Zn	5.84	3.66	9.64	1.43
Fe	18.58	6.38	36.81	8.04

**Table 3:** Mean concentration (mg/kg^−1^) of 13 metals in roe deer tumours according to sex.

	Hg			Cd			Cr			Mn		
	Min	Mean	Max	Min	Mean	Max	Min	Mean	Max	Min	Mean	Max
♀	0.000	0.017	0.044	0.000	0.027	0.160	0.175	1.145	19.600	0.249	0.993	2.500
♂	0.000	0.020	0.096	0.000	0.029	0.200	0.200	0.835	2.740	0.228	1.205	2.630
	Al			Co			Cu			Ni		
	Min	Mean	Max	Min	Mean	Max	Min	Mean	Max	Min	Mean	Max
♀	0.134	1.493	6.060	0.050	0.173	2.400	0.056	1.100	4.360	0.000	4.862	18.900
♂	0.281	1.635	2.960	0.000	0.178	2.250	0.184	1.167	2.830	0.000	6.690	27.600
	Se			Zn			Fe			Pb		
	Min	Mean	Max	Min	Mean	Max	Min	Mean	Max	Min	Mean	Max
♀	0.015	0.257	0.787	3.660	5.570	9.640	6.380	26.665	29.710	0.006	0.001	0.050
♂	0.000	0.383	0.880	4.120	6.090	9.080	7.650	20.775	36.810	0.005	0.019	0.070
	As											
	Min	Mean	Max									
♀	0.005	0.005	0.006									
♂	0.005	0.005	0.008									

**Table 4:** Mean concentration (mg/kg^−1^) of each metal according to papilloma type. P = pedunculate, S = sessile.

Papiloma Type	Hg			Cd			Cr			Mn		
	Min	Mean	Max	Min	Mean	Max	Min	Mean	Max	Min	Mean	Max
S	0.000	0.015	0.064	0.000	0.029	0.200	0.175	1.218	19.600	0.228	1.095	2.630
P	0.000	0.023	0.096	0.000	0.027	0.099	0.183	0.719	1.630	0.316	1.109	2.510
	Al			Co			Cu			Ni		
	Min	Mean	Max	Min	Mean	Max	Min	Mean	Max	Min	Mean	Max
S	0.134	1.549	6.060	0.000	0.439	2.400	0.179	1.165	4.360	0.000	5.976	19.100
P	0.163	1.585	2.700	0.055	0.194	0.930	0.056	1.099	2.830	0.000	5.595	27.600
	Se			Zn			Fe			*Pb*		
	Min	Mean	Max	Min	Mean	Max	Min	Mean	Max	Min	Mean	Max
S	0.000	0.312	0.823	3.660	5.808	9.640	6.380	26.415	263.100	0.006	0.014	0.070
P	0.000	0.333	0.880	3.780	5.869	9.170	7.280	20.394	36.810	0.005	0.016	0.060
	*As*											
	Min	Mean	Max									
S	0.005	0.005	0.008									
P	0.005	0.005	0.008									

## Data Availability

All data are available upon request of any author.
